# Flexible Theoretical Calculation of Loop Length and Area Density of Weft-Knitted Structures: Part I

**DOI:** 10.3390/ma14113059

**Published:** 2021-06-03

**Authors:** Edgaras Arbataitis, Daiva Mikucioniene, Liudmyla Halavska

**Affiliations:** 1 Department of Production Engineering, Kaunas University of Technology, 44249 Kaunas, Lithuania; edgaras.arbataitis@ktu.edu; 2 Department of Textile Technology and Design, Kyiv National University of Technologies and Design, 01011 Kyiv, Ukraine; galavska.ly@knutd.edu.ua

**Keywords:** loop length, area density, weft-knitted structure, geometrical model

## Abstract

This work presents a simple and flexible method for theoretical calculation of the main structural parameter of weft-knitted fabrics’—the loop length and one of the main characteristics of textile fabrics—area density, which combines physical and economical aspects. It helps to predict many physical properties and the mechanical behaviour, which is especially important for protective textiles, and allows predicting potential yarn consumption for knitting of one square meter of the fabric. The main idea of the proposed method, based on Čiukas geometrical model, is to calculate different parts of the knitted loop separately, which gives a great flexibility of such modelling. The proposed theoretical formulas can be used for various weft-knitted structures, give very low errors to empirical calculations, and are easy to use. It is a big advantage because known geometric models only allow a loop length of some particular pattern to be calculated, usually of single jersey or rib 1 × 1.

## 1. Introduction

The aim of theoretical designing of knitted fabrics—having certain a knitting machine and a vision to produce a high-quality, aesthetic and economical knit—is to select yarns of appropriate fibre composition, structure and properties, knitting pattern and theoretically to calculate the loop length and area density of the fabric by mathematical formulas. It is especially important for knitted fabrics that are going to be used for protective garments in order to predict their physical properties and mechanical behaviour during external hazardous factors [1]. In almost 100 years, many attempts have been made and various models have been proposed by Dalidovich [2], Pierce [3], Chamberlain [4], Doyle [5], Leaf [6,7], Munden and Postle [8,9,10], Čiukas [11,12], Kurbak and others [13,14,15,16] to obtain relationships between dimensions of weft-knitted fabrics, properties of the constituent yarns as well as variable factors in knitting, and to describe the knitted structure by mathematical formulas in order to predict the structural and physical properties of the knitted fabric before knitting. The physical and mechanical properties of the weft-knitted fabrics, such as breaking characteristics, dimensional stability, air permeability, etc., are highly dependent on the average loop length in the pattern repeat of the fabric, i.e., on the length of the yarn in one unit of knitted structure [1,17]. While yarn properties, such as raw material, spinning system, linear density and twist [18], and relaxation state [19,20] have significant influence on the geometry of the knitted loop.

In general, there are three types of models used for theoretical designing: geometrical, based on the loop geometry; mechanical, based not only on the geometry but also on forces acting in the yarn bent into the loop and derived from equilibrium considerations of the forces and couples applied to one loop by its neighbours; and energetic, based on the minimum of energy required to obtain particular form of the loop. It is suggested that the natural shape of the knitted loop is determined by minimum energy conditions, meaning that the loop tends to its relaxed state [10,21,22,23]. Usually, in order to simplify the modelling and mechanical models themselves, an assumption is made that the knitted loop is in fact flat (two-dimensional) and its shape is a function of the forces acting in the plane of the fabric [7]. However, for practical purposes in theoretical designing of various weft-knitted patterns, the geometrical models are more suitable than the mechanical or energetic models as it does not require to know beforehand exact values of mechanical forces acting in the loop and between the adjacent loops.

Geometrical models of the weft-knitted structure are used to establish interdependence of the loop length, density of stitches and the yarn diameter. The geometrical description of a yarn bent into a knitted loop includes the option to describe the axial line of the yarn and changes in the shape and diameter of the yarn along this axial line [24,25,26]. In order to simplify geometry of the loop and calculation of the loop length, geometrical models are based on the following assumptions:

- The plane projection (two-dimensional form) of the yarn, bent into the loop, coincides with its three-dimensional form;
- The yarn bent into the loop has a cylindrical shape; its diameter over the entire length is circular and constant;
- The yarn elasticity over the entire length is constant;
- The loop length is equal to the length of the yarn axis.

There are several variants of the plain knitted loop geometry proposed for the mathematical calculation of the loop length, on which further investigations were based. In Chamberlain’s model (1926), the loop consists of parts of circles joined by straight lines [4]; in Pierce’s model (1947), it is embraced that the top arc (loop head) and the bottom half-arcs (loop feet) of the loop are composed of arcs and straight segments [3]; in Dalidovitch’s model (1928) [2] and the Kurbak model (1970) [13,15], which are the closest to the real geometry of the plain-knitted loop, the loop is composed of the top arc (loop head), two straight segments (loop legs) and two bottom half-arcs (loop feet). However, the presented models generally can be used only for modelling of a single jersey knitted loop, usually made from a woollen yarn. Kurbak [13,15,16,23] developed geometrical models for various weft-knitted structures, such as rib 1 × 1, purl 1 × 1, Milano rib; however, for each pattern, different formulas are used, which require initial geometrical data.

**Figure 1:**
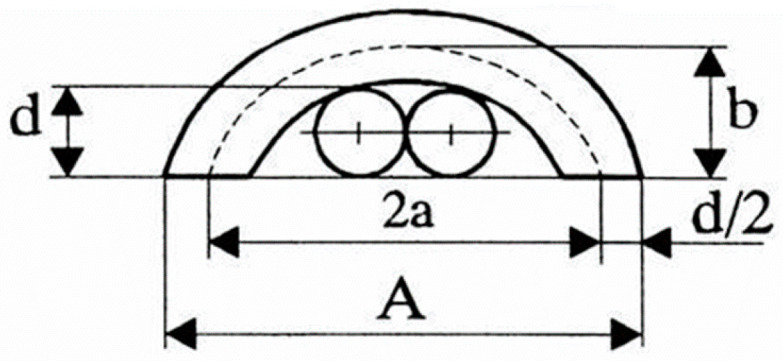
Geometrical model of the loop head by Čiukas (here *d*—yarn diameter; *A*—wale spacing; *a*—large radius of ellipse; *b*—small radius of ellipse), (mm).

After deep investigations into the possible real geometry of the weft-knitted loop, in the new interpretation of the loop geometry proposed by Čiukas [11,12], it was established that the top arc of the loop has an elliptical shape instead of a circle (as in the most cases of geometrical models) (Figure 1), and the loop length comprises of the needle loop length (the sum of lengths of the head and legs of the loop) and the length of float, that joins two adjacent loops in one course and can be of different length (depending on how many needles are missed between two stitches and if the stitches are made on the same needle bar or not [11,26]).

According to Figure 1, the perimeter of the ellipse *PE* is:

(1) $$PE=\pi \left(a+b\right)$$

where *a* is the large radius of ellipse; *b* is the small radius of ellipse.

According to the given geometry of the loop head, the radius *a* and *b* can be expressed by Formulas (2) and (3), accordingly:

(2) $$a=\frac{1}{2}\left(A-d\right);$$

(3) b=d+0.5d=1.5d;

where *A* is the wale spacing, in mm; *d* is the yarn diameter, in mm.

In such case the length of the loop head *l_lh_*, which coincide with the half of perimeter of the ellipsis *PE*, can be expressed by Formula (4) [26]:

(4) $$l_{lh}=0.5\pi \left(0.5\left(A-d\right)+1.5d\right)=0.5\pi \left(0.5A+d\right);$$

The aim of this research was to adapt theory of Čiukas’ geometrical modelling to basic weft-knitted patterns, such as rib structures with four different pattern repeats and two variants of purl knit structures, and to propose experimentally proven mathematical formulas which can be flexibly applied for theoretical calculation of the loop length and area density.

## 2. Materials and Methods

**Figure 2:**
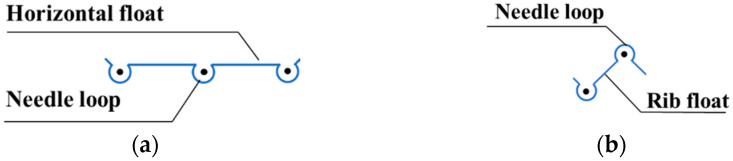
Graphical notation of the needle loop, (**a**) horizontal float and (**b**) rib float.

According to the theory of Čiukas [11,12,26], the weft-knitted loop is comprised of the needle loop (the loop head plus two legs) and a float, which connects two adjacent needle loops and can be analysed separately for every different pattern. The weft-knitted float can be characterized as the horizontal float, if it joins two adjacent needle loops formed on the same needle bar, or the rib float, if it joins two adjacent needle loops formed on the opposite needle bars (see in Figure 2).

The length of the needle loop formed on one-needle bar knitting machine is calculated according to the Formula (5) [11,12], while the length of the needle loop formed on two-needle bar knitting machine is calculated according to the Formula (6) [26]:

(5) $$l_{s}=0.5\pi \left(0.5A+d\right)+2B;$$

(6) $$l_{d}=0.5\pi \sqrt{0.25A^{2}+Ad+2d^{2}}+2\sqrt{B+2d};$$

where *l_s_* is the length of the needle loop made on a single needle bar, in mm; *l_d_* is the length of the needle loop made on a double needle bar, in mm; *A* is the wale spacing, in mm; *B* is the course spacing, in mm; *d* is the yarn diameter, in mm.

According to Formula (5), projection of the loop, knitted on one-needle bar knitting machine, in the plain, in fact, is equal to the factual loop length. Although further investigation showed that this assumption is not correct in the case of loops formed on two needle-bar knitting machines. It led to the conclusion that for the rib needle loop length calculations spatial position and shape of the loop must be considered and taken into account.

The length of the horizontal float of any length is calculated according to the Formula (7), and the length of the rib float of any length is calculated according to the Formula (8) [26]:

(7) $$l_{h}=0.5\pi \left(0.25iA+d\right);$$

where *i* is the dimensionless index describing the length of the horizontal float and it can have only even values: 2—for the shortest float, which joins two needle loops on the adjacent needles, 4—for the float when one needle is missed between two needle loops, 6—for the float when two needles are missed, etc.; *A* is the wale spacing, in mm; *d* is the yarn diameter, in mm.

(8) $$l_{r}=0.5\pi \left(0.5\sqrt{{\left(0.5iA-d\right)}^{2}+9d^{2}}+1.5d\right);$$

where *i* is the dimensionless index describing the length of the rib float and it can have only uneven values: 1—for the shortest rib float, which joins two needle loops on the adjacent needles in two needle bars, 3—for the float when one needle is missed in both needle bars, 5—for the float when two needles are missed in both needle bars, etc.; *A* is the wale spacing, in mm; *d* is the yarn diameter, in mm.

In general case, area density of a weft-knitted fabric is calculated by Formula (9) [12]:

(9) $$M=\frac{LY\cdot T}{A\cdot B\cdot R\cdot H};$$

where *LY* is the total length of the yarn in the pattern repeat (in mm), which is the sum of the lengths of all elements, i.e., all needle loops and floats in the pattern repeat; *T* is linear density of the yarn, in tex; *A* is the wale spacing, in mm; *B* is the course spacing, in mm; *R* and *H* is the size of the pattern repeat in horizontal and vertical directions, respectively.

To find the average value of the loop length $\bar{l}$ in a pattern repeat, the total length of the yarn in the pattern repeat *LY* is divided by the number of loops in the pattern repeat.

**Figure 3:**
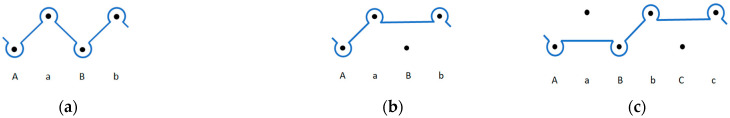

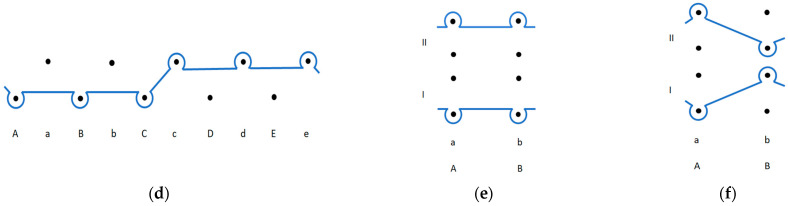
Graphic notation of the investigated structures: (**a**) rib 1 × 1; (**b**) rib 1 × 2; (**c**) rib 2 × 2; (**d**) rib 3 × 3; (**e**) purl 1 × 1; (**f**) moss-stitch purl.

Experimental samples were produced in four variants of rib structure and two variants of purl knit structure (presented in Figure 3) on an electronic flat weft knitting machine Shima Seiki SES 122 (Japan), gauge E12. The samples were knitted from original yarn packages and no additional lubrication during knitting was used. All samples were knitted from two ply blended 50% wool/50% acrylic yarns with linear density 40 tex × 2.

Structural parameters of the experimental knits were measured according to Standard BS 5441:1998 and are presented in Table 1. All knitted fabrics were investigated in a “grey” state, i.e., without finishing, but after 1-week relaxation in a free state in standard atmospheric conditions at 20 ± 2 °C temperature and 65 ± 4% humidity according to Standard EN ISO 139:2005.

## 3. Results and Discussions

### 3.1. Theoretical Calculation of Weft-Knitted Loop Length

To apply the model to a specific pattern and calculate lengths of specific structural elements, firstly, type and number of the elements in the specific knitting pattern repeat must be determined. According to the graphical notation of investigated knitted structures (see in Figure 3), structural elements and their number in knitting pattern repeat are presented in Table 2.

Needle loops in Rib 1 × 1, Rib 1 × 2 and Rib 2 × 2 patterns are the same in size, as all of them are formed on two-needle bars and have at least one adjacent rib float (see in Figure 3). In order to calculate the average loop length of these knits, lengths of individual elements, i.e., needle loops and connecting floats, have to be calculated according to Formulas (6)–(8), depending on the type of the element.

In Rib 3 × 3 structure, there is an extra plain loop inserted between two segments of rib 1 × 1 (see in Figure 3). The loop geometry and length of an extra plain loop differ from a rib 1 × 1 segment because this loop is connected to the adjacent loops by horizontal floats. Therefore, the wale spacing and needle loop length of the rib 1 × 1 and plain knit segments are different. It was also stated by Čiukas in [26] that if several loops are formed on a front needle bar and several loops on a back needle bar consecutively, it will change the wale spacing and the width of the knitted fabric. The wider needle loop is the one that connects at least on one side to the needle loop in the opposite needle bar via rib float. In such a case, the new wale spacing A’ for wider loops can be calculated by Formula (10) [26]:

(10) $$A^{\prime }=A\frac{2K+1}{K+1};$$

where *A* is the wale spacing, in mm; *K* is the number of reverse loops in the pattern repeat. In this case, for Rib 3 × 3: $A^{\prime }=1.05$ mm.

From this point, calculations are continued according to the proposed geometrical model: the length of one-bar needle loop and the horizontal float is calculated by Formulas (5) and (7) accordingly, using original wale spacing *A*. The length of two-bars needle loop and the rib float are calculated by Formulas (6) and (8) accordingly, using newly calculated wale spacing *A’* instead of *A*. The results of calculations are presented in Table 3.

To calculate the average loop length $\bar{l}$ in a pattern repeat, the total length of the yarn in the pattern repeat *LY* is divided by the number of loops in the pattern repeat. General expression for the total yarn length consumed in a pattern repeat can be expressed by Formula (11):

(11) $$LY=N_{ls}\cdot l_{s}+N_{ld}\cdot l_{d}+N_{lh}\cdot l_{h}+N_{lr}\cdot l_{r};$$

where $N_{ls}$ is the number of one-needle bar needle loops; *l_s_* is the length of one-needle bar needle loop in mm; $N_{ld}$ is the number of two-needle bar needle loops; *l_d_* is the length of two-needle bar needle loop in mm; $N_{lh}$ is the number of horizontal floats; *l_h_* is the length of horizontal float in mm; $N_{lr}$ is the number of rib floats; *l_r_* is the length of rib float in mm.

In the case of patterns Rib 1 × 1, Rib 1 × 2, Rib 2 × 2 and Rib 3 × 3, following expressions will be used for calculation of the total yarn length in the pattern repeat and the average loop length in the pattern repeat:

(12) $$LY_{Rib1\times 1}=2l_{d}+2l_{r};$$

(13) $${\bar{l}}_{Rib1\times 1}=\frac{LY_{Rib1\times 1}}{2l_{d}};$$

(14) $$LY_{Rib1\times 2}=3l_{d}+1l_{h}+2l_{r};$$

(15) $${\bar{l}}_{Rib1\times 2}=\frac{LY_{Rib1\times 2}}{3l_{d}};$$

(16) $$LY_{Rib2\times 2}=4l_{d}+2l_{h}+2l_{r};$$

(17) $${\bar{l}}_{Rib2\times 2}=\frac{LY_{Rib2\times 2}}{4l_{d}};$$

(18) $$LY_{Rib3\times 3}=2l_{s}+4l_{d}+4l_{h}+2l_{r};$$

(19) $${\bar{l}}_{Rib3\times 3}=\frac{LY_{Rib3\times 3}}{2l_{s}+4l_{d}}.$$

In the case of Purl 1 × 1 and Moss-stitch purl knits the calculations of the length of the structural elements are based on the same theory as for single jersey and rib structures accordingly. Each knit is comprised of identical but opposite to each other courses of loops. Purl 1 × 1 knit is comprised of two single jersey courses, one of which is being formed on the front needle bar and the second one on the back needle bar (see in Figure 3). In Moss-stitch purl knit pattern repeat, two courses of rib 1 × 1 are used as opposite to each other in turn (see in Figure 3).

For Purl 1 × 1, the length of one-bar needle loop and the horizontal float is calculated by Formulas (5) and (7) accordingly. The lengths of the Moss-stitch purl knit structural elements can be calculated by Formulas (6) and (8). Total length of the yarn in the pattern repeats and the average length of loop in a pattern repeat are calculated by following expressions:

(20) $$LY_{Purl1\times 1}=2l_{s}+2l_{h};$$

(21) $${\bar{l}}_{Purl1\times 1}=\frac{LY_{Purl1\times 1}}{2_{ls}};$$

(22) $$LY_{Moss-stitch}=4l_{d}+4l_{r};$$

(23) $${\bar{l}}_{Moss-stitch}=\frac{LY_{Moss-stitch}}{4_{ld}};$$

Results of the total yarn length in the pattern repeat and the average loop length in the pattern repeat for all knits are presented in Table 4.

In order to analyse the accuracy of the theoretical calculations, the relative error values between the theoretically calculated and experimentally measured (see in Table 1) results were calculated. As it can be seen from the results presented in Table 4, the accuracy of theoretical calculations of the average loop length in comparison to the experimentally measured data is very high in all cases, as the error did not exceed 4% for all analysed rib structures and purl knits. Thus, it demonstrates that presented model and theoretical Formulas can be used for modelling of the loop length of rib and purl structures of various pattern repeats, as it gives very high accuracy.

### 3.2. Theoretical Calculation of Weft-Knitted Fabric Area Density

The next step of the application of the geometrical model is theoretical calculation of the area density. For all analysed rib structures and Moss-stitch purl knit the given Formula (9) was used. While investigating Purl 1 × 1 knit, it was noticed that the loop shape changes its form. All loops of the entire row are formed either on front or back needle bar. Each next row is being formed on the opposite needle bar than the previous one. This force loops to bend over each other and overlap. Only top arcs of the needle loop (loop heads) and bottom arcs of the sinker loops (loop feet) are visible on the surface of the knitted fabric. This overlapping of the loops must be considered. According to the geometrical model proposed in [11,12], the height of the needle loop top arc is the same as of the sinker arc and is equal to 1.5 *d.* Visual investigation of the fabric showed that two rows are covered by only one needle arc and one sinker arc. Therefore, the following expression can be used to calculate the area density of the Purl 1 × 1 knit:

(24) $$M_{Purl1\times 1}=\frac{LY\cdot T}{A\cdot B\cdot R\cdot H\cdot 1.5\cdot d};$$

where *LY* is the total length of the yarn in the pattern repeat, in mm; *T* is the linear density of the yarn, in tex; *A* is the wale spacing, in mm; *B* is the course spacing, in mm; *R* and *H* is the size of the pattern repeat in horizontal and vertical directions, respectively; *d* is the yarn diameter, in mm.

Vertical *H* and horizontal *R* pattern repeats can be determined from the graphic representation of knits (Figure 3). Vertical and horizontal repeats are: for Rib 1 × 1: *H* = 1 and *R* = 2; for Rib 1 × 2: *H* = 1 and *R* = 3; for Rib 2 × 2: *H* = 1 and *R* = 4; for Rib 3 × 3: *H* = 1 and *R* = 6; for Purl 1 × 1: *H* = 2 and *R* = 1; for Moss-stitch purl: *H* = 2 and *R* = 2.

Results of the area density calculations are presented in Table 5.

The relative error between the theoretically calculated and experimentally measured values (presented in Table 5) demonstrate very good accuracy of the presented formula for area density calculation, as the relative error is very low, in almost all cases lower than 3%. Thus, it can be recommended for various rib and purl structured knitted fabrics.

## 4. Conclusions

The presented work demonstrates that mathematical formulas for the loop length and area density theoretical calculation, developed according to Čiukas geometrical model, give very good accuracy for various rib and purl structured knits. The relative error of theoretical calculation of the loop length of rib and purl structured knits with different pattern repeats did not exceed 4% and in most cases was lower than 3%. The relative error of theoretical calculation of the area density of investigated knits did not exceed 3%. It is very important that presented formulas for theoretical calculation of the loop length and area density are simple to use and can be applied for different pattern repeats of the rib and purl structures. It helps to predict many physical properties and the mechanical behaviour, which is especially important for protective textile, and allows to predict the possible consumption of yarns for knitting of one square meter of the fabric. It is a big advantage in comparison to other known geometrical models that are developed for specific structures, generally for single jersey or rib 1 × 1.

Based on the findings of this study, future work will focus on experimental approvement of this model to complex fancy and combined weft-knitted structures with tucks and tuck-stitches in a pattern to show the flexibility of the model.

## Figures and Tables

**Table 1:** Structural parameters of the knits.

Pattern.	Yarn Diameter *d*, mm	Wale Density *P_w_,* cm^−1^	Course Density *P_c_,* cm^−1^	Wale Spacing *A,* mm	Course Spacing *B*, mm	Loop Length *l*, mm	Area Density *M,* g/m^2^
Rib 1 × 1	0.4	11.3 ± 0.2	8.0 ± 0.1	0.88	1.25	6.1 ± 0.2	449.8 ± 4
Rib 1 × 2		11.0 ± 0.2	8.2 ± 0.2	0.91	1.22	6.2 ± 0.2	424.6 ± 4
Rib 2 × 2		12.8 ± 0.2	8.4 ± 0.1	0.78	1.19	5.7 ± 0.2	491.2 ± 4
Rib 3 × 3		16.7 ± 0.2	8.5 ± 0.2	0.60	1.18	5.5 ± 0.3	618.0 ± 5
Purl 1 × 1		5.1 ± 0.1	5.3 ± 0.3	1.96	1.89	7.8 ± 0.3	292.0 ± 6
Moss-stitch purl		5.3 ± 0.2	10.2 ± 0.2	1.89	0.98	6.7 ± 0.2	287.1 ± 5

**Table 2:** Number of structural elements in pattern repeat.

Pattern	Number of One-Needle Bar Needle Loops *N_ls_*	Number of Two-Needle Bar Needle Loops *N_ld_*	Number of Horizontal Floats *N_lh_*	Number of Rib Floats *N_lr_*
Rib 1 × 1	-	2	-	2
Rib 1 × 2	-	3	1	2
Rib 2 × 2	-	4	2	2
Rib 3 × 3	2	4	4	2
Purl 1 × 1	2	-	2	-
Moss-stitch purl	-	4	-	4

**Table 3:** Theoretically calculated lengths of structure elements.

Pattern	One-Bar Needle Loop *l_s_*, mm	Two-Bars Needle Loop *l_d_*, mm	Horizontal Float *l_h_*, mm	Rib Float *l_r_*, mm
Rib 1 × 1	-	4.33	-	1.88
Rib 1 × 2	-	4.34	1.36	1.89
Rib 2 × 2	-	4.21	1.24	1.88
Rib 3 × 3	3.45	4.39	1.10	1.89
Purl 1 × 1	5.94	-	2.17	-
Moss-stitch purl	-	4.87	-	1.98

**Table 4:** Theoretically calculated yarn length in the pattern repeat, average loop length and relative error between the theoretically calculated and experimentally measured values.

Pattern	Yarn Length in the Pattern Repeat *LY*, mm	Average Loop Length in the Pattern Repeat $\bar{l}$, mm	Relative Error between Theoretical and Experimental Values, %
Rib 1 × 1	12.42	6.21	1.80
Rib 1 × 2	18.16	6.05	2.42
Rib 2 × 2	23.10	5.78	1.40
Rib 3 × 3	32.64	5.44	1.09
Purl 1 × 1	16.22	8.11	3.97
Moss-stitch purl	27.38	6.85	2.24

**Table 5:** Area density and relative error between the theoretically calculated and experimentally measured values.

Pattern	Theoretically Calculated Area Density *M,* g/m^2^	Experimentally Measured Area Density, g/m^2^	Relative Error between Theoretical and Experimental Values, %
Rib 1 × 1	449.28	449.8 ± 4	0.12
Rib 1 × 2	424.84	424.6 ± 4	0.06
Rib 2 × 2	496.78	491.2 ± 4	1.14
Rib 3 × 3	617.81	618.0 ± 5	0.03
Purl 1 × 1	292.21	292.0 ± 6	0.07
Moss-stitch purl	296.05	287.1 ± 5	3.12

## Data Availability

Data sharing is not applicable to this article.
